# Several implications for the pathogenesis and treatment of thrombosis in PNH patients according to multiomics analysis

**DOI:** 10.1186/s12967-024-04936-y

**Published:** 2024-02-03

**Authors:** Yingying Chen, Xiaorui Meng, Yinxing Wang, Chunyan Liu, Rong Fu

**Affiliations:** 1 Department of Hematology, Tianjin Medical University General Hospital, 154 Anshan Street, Tianjin, 300052 People’s Republic of China; 2 Tianjin Key Laboratory of Bone Marrow Failure and Malignant Hemopoietic Clone Control, Tianjin, China; 3 School of Biomedical Sciences, LKS Faculty of Medicine, The University of Hong Kong, Hong Kong, China

**To the editor:**

Thrombosis is the most frequent complication and the main cause of death in paroxysmal nocturnal hemoglobinuria (PNH) patients. Recently, we admitted two cases of PNH patients with acute thrombotic events who developed new pulmonary embolism/portal vein thrombus despite anticoagulant therapy (Additional file 1: Table S1). Peripheral blood was collected from these two patients (PT group) for whole exome sequencing (WES), single-cell RNA sequencing (scRNA-seq), and iTRAQ-based proteomics, and the results were compared with those of corresponding tests for PNH patients without thrombotic events (P group) (Additional file 1: Materials & Methods).

Through WES, we identified 17 mutation loci that existed only in both two patients of PT group, involving 14 genes: ZNF471, SLC35G4, REG3A, MUC17, ASB1, AHNAK2, MAP3K2, ANKRD36, KCNH6, MUC19, HCAR3, MUC4, KRTAP10-12, and DNAH3 (Additional file 1: Table S2). Among them, MUC4 was shown to be associated with PNH thrombosis in our previous work [1]; ANKRD36 has been reported to be related to thrombosis in patients with thrombotic thrombocytopenic purpura [2]. Mutations in other genes have not been clearly reported to be associated with thrombosis, but mainly associated with cell adhesion, cytoskeleton, and leukocyte migration (Fig. 1A). Although the role of these gene mutations in PNH thrombosis still needs to be further validated, we recommend that prophylactic anticoagulant therapy should be given to PNH patients who have gene mutations strongly associated with thrombosis.

**Fig. 1:**
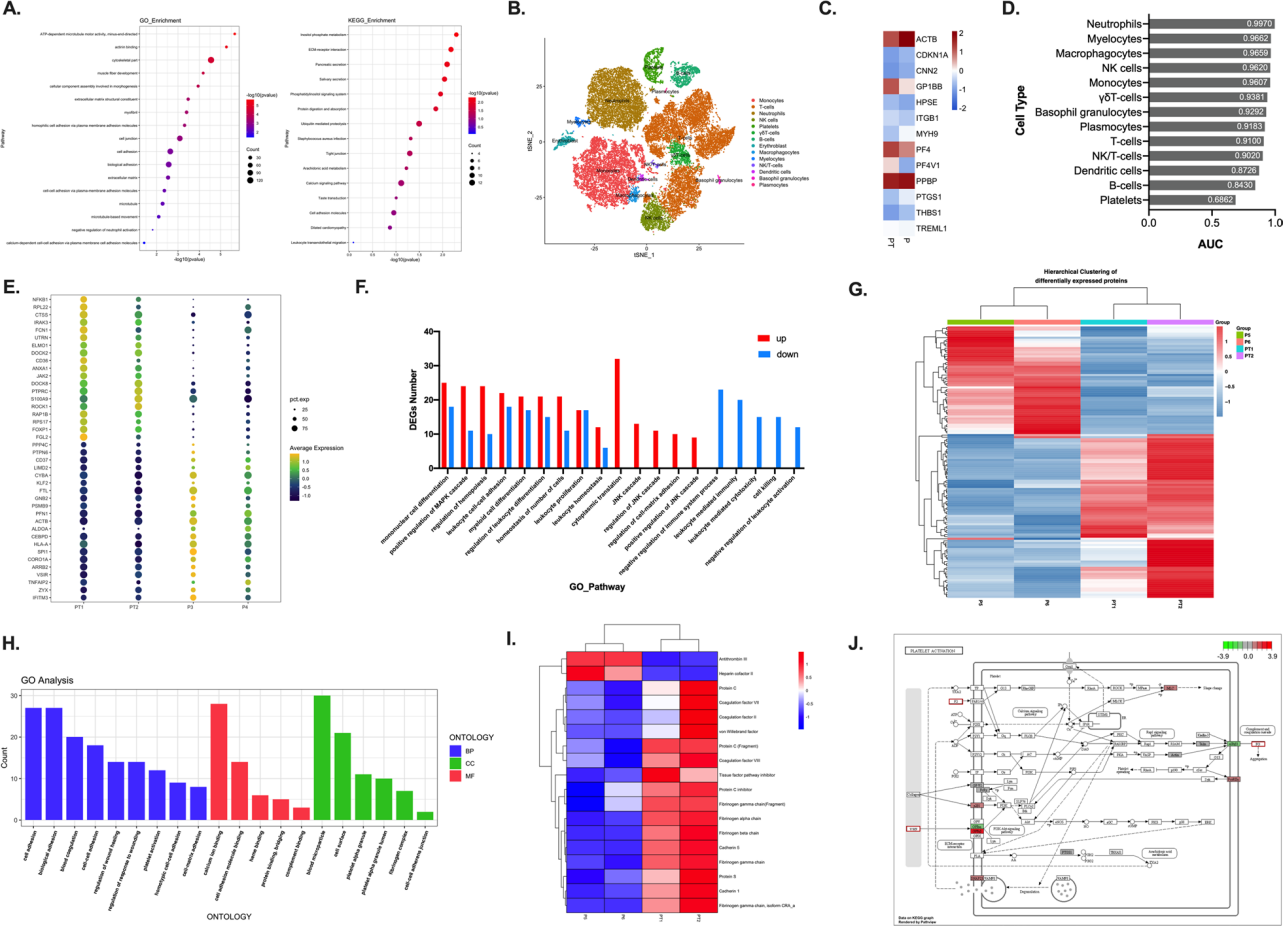
Results of multiomics analysis. **A** GO enrichment analysis (left) and KEGG enrichment analysis (right) of mutated genes in the PT group. The color of the bubble indicates the -log10 (p value) of the GO term or KEGG pathway, and the size of the bubble signifies the number of genes associated with a term. **B** t-SNE representing 35,949 cells from 14 types of peripheral blood. **C** Heatmap displaying expression levels of 13 target genes in platelets from the two groups. **D** Prioritization of cell types related to thrombosis using Augur. A high area under the curve (AUC) indicates high sensitivity of the cells to changes in thrombosis. **E** Dot plot showing expression levels of differentially expressed genes (DEGs) in myeloid cells from the two groups. **F** Number of DEGs associated with a term from GO enrichment analysis of myeloid cells. **G** Heatmap displaying expression levels of differentially expressed proteins (DEPs) from the two groups. **H** GO enrichment analysis of DEPs between the PT and P groups. **I** Heatmap displaying the protein expression level of 18 DEPs in four samples. **J** Pathway diagram of platelet activation. The red border represents upregulated plasma proteins in the PT group, and the red/green filled box represents upregulated/downregulated genes in the PT group

Next, we analyzed the changes in transcription levels of the two groups by scRNA-seq. A total of 35,949 cells were included in analysis and were divided into 14 cell types (Fig. 1B, Additional file 1: Figure S1A). We first analyzed the transcriptomic changes in PLT and identified 268 differentially expressed genes (DEGs) between the two groups (Additional file 1: Figure S1B). These genes are mainly involved in blood coagulation, platelet activation and aggregation (Fig. 1C, Additional file 1: Figure S1C). PF4/PF4V1, which has been reported promote platelet aggregation by activating platelet in vitro [3], were upregulated in the PT group. In addition, we found that expression of PLT contractile genes (CNN2/ACTB/MYH9) was downregulated. Reduced platelet contractile function making the thrombus more prone to detachment, which may be related to new pulmonary embolism/portal vein thrombosis in the two patients during anticoagulant therapy. Through Augur analysis, we found that myeloid cells, including neutrophils, myelocytes and monocytes, contributed significantly to the thrombotic phenotype (Fig. 1D). Therefore, we analyzed the DEGs of myeloid cells and found that genes related to leukocyte activation, adhesion and migration to the endothelium were upregulated, while genes related to leukocyte-mediated immunity and cell killing were downregulated (Fig. 1E, F). In general, we can specifically discover the role of different cell types in thrombosis in PNH patients through scRNA-seq. On transcriptional level, aggregation and activation of platelets, adhesion and migration of myeloid cells play important roles in PNH thrombosis.

Finally, iTRAQ-based proteomics was used to quantitatively detect peripheral blood plasma proteins. A total of 79 upregulated and 52 downregulated proteins were detected in the PT group (Fig. 1G, Additional file 1: Figure S1D). Enrichment analysis shows that the differentially expressed proteins (DEPs) are involved in cell adhesion, platelet activation, fibrinogen complex, and complement and coagulation cascade (Fig. 1H, Additional file 1: Figure S1E). Plasma levels of procoagulant substances, including prothrombin, coagulation factor VII/VIII, von Willebrand factor (vWF), and fibrinogen, were significantly increased in the PT group, while the levels of antithrombin III (AT III) and heparin cofactor (HC II) were decreased, suggesting platelet activation and coagulation activation (Fig. 1I). Acquired AT III deficiency is a common cause of heparin resistance [4], and the decrease in AT III levels in these two patients may be related to the widespread thrombotic events that occurred even after the standard dose of heparin anticoagulant therapy. Interestingly, we also found that levels of the protein S, protein C, tissue factor pathway inhibitor (TFPI) and protein C inhibitor (PCI) was upregulated, which we believe is a protective response of the body against excessive coagulation and thrombosis. The results of scRNA-seq demonstrated activation of platelet function in thrombotic patients, and proteomics further showed that increased plasma levels of vWF, prothrombin, and fibrin, promoting platelet activation and aggregation, which is closely related to thrombosis in PNH patients (Fig. 1J).

In conclusion, we demonstrate that platelet activation, coagulation cascades, and leukocyte cell adhesion are closely related to thrombosis in PNH patients at multiple levels of gene, transcription, and protein. Due to the limited number of cases, thrombosis-related factors in PNH patients still need to be further studied. We recommend that patients with PNH be screened for mutation after diagnosis and that patients at high risk of thrombosis be treated with prophylactic anticoagulant therapy. The observed decreases in platelet systolic function and AT III level may be related to the poor anticoagulant effect of heparin in some PNH patients with thrombosis. Monitoring AT III levels and adjusting heparin dose accordingly during anticoagulant therapy may improve the efficacy and safety of treatment. Platelet activating factors such as PF4V1/PF4 are also expected to become new targets for future PNH thrombosis treatment.

### Supplementary Information

**Additional file 1: Materials & Methods. Figure S1.** A. Dot plot displaying the expression level of marker genes in 14 clusters. B. Volcano plot showing differentially expressed genes (DEGs) across platelets in the PT and P groups. A total of 268 DEGs were identified using the Wilcoxon rank sum test with an absolute log2-fold change (FC) ≥ 0.5 and a p value < 0.05, including 193 upregulated genes in the PT group and 75 upregulated genes in the P group. C. GO enrichment analysis of DEGs in platelets. The color of the bubble indicates the Bonferroni-adjusted p value of the GO term, and the size of the bubble signifies the GeneRatio associated with a term. D. Volcano plot showing differentially expressed proteins (DEPs) between the PT and P groups. A total of 131 DEPs were identified using a fold change > 1.5 and a Q value < 0.05, including 79 upregulated proteins in the PT group and 52 upregulated proteins in the P group. E. KEGG enrichment analysis of DEPs. The color of the bubble indicates the -log10 (p value) of the KEGG terms, and the size of the bubble signifies the GeneRatio associated with a term. **Table S1.** Clinical characteristics of 8 PNH patients. **Table S2.** Annotation information of mutant gene in PT group.

## Data Availability

The data that support the findings of this study are available in Bioproject at https://dataview.ncbi.nlm.nih.gov/object/PRJNA1061334?reviewer=vcp6n6o6nuvu7tirnrh790tuqh.
