# Supplementary material for: Several implications for the pathogenesis and treatment of thrombosis in PNH patients according to multiomics analysis
Source: J Transl Med. 2024 Feb 3;22:129. doi: 10.1186/s12967-024-04936-y (PMC10837891; doi:10.1186/s12967-024-04936-y)
Supplement: Supplementary file 1 — Additional file 1: Materials & Methods. Figure S1. A. Dot plot displaying the expression level of marker genes in 14 clusters. B. Volcano plot showing differentially expressed genes (DEGs) across platelets in the PT and P groups. A total of 268 DEGs were identified using the Wilcoxon rank sum test with an absolute log2-fold change (FC) ≥ 0.5 and a p value < 0.05, including 193 upregulated genes in the PT group and 75 upregulated genes in the P group. C. GO enrichment analysis of DEGs in platelets. The color of the bubble indicates the Bonferroni-adjusted p value of the GO term, and the size of the bubble signifies the GeneRatio associated with a term. D. Volcano plot showing differentially expressed proteins (DEPs) between the PT and P groups. A total of 131 DEPs were identified using a fold change > 1.5 and a Q value < 0.05, including 79 upregulated proteins in the PT group and 52 upregulated proteins in the P group. E. KEGG enrichment analysis of DEPs. The color of the bubble indicates the -log10 (p value) of the KEGG terms, and the size of the bubble signifies the GeneRatio associated with a term. Table S1. Clinical characteristics of 8 PNH patients. Table S2. Annotation information of mutant gene in PT group. [file 12967_2024_4936_MOESM1_ESM.pdf]

Supplemental Material

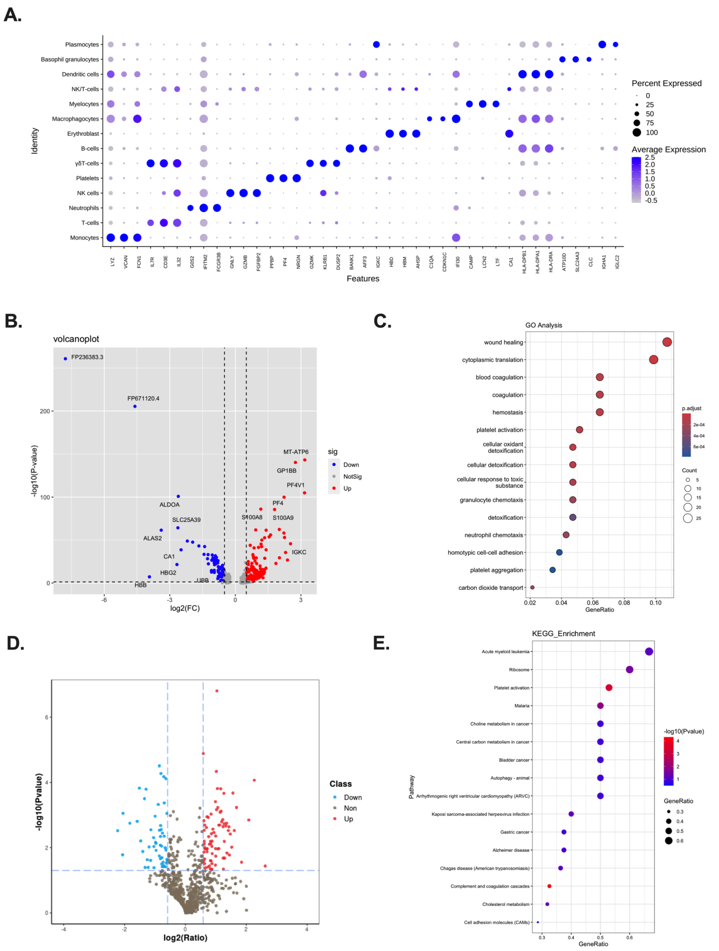

Fig.S1. A. Dot plot displaying the expression level of marker genes in 14 clusters. B. Volcano plot showing differentially expressed genes (DEGs) across platelets in the PT and P groups. A total of 268 DEGs were identified using the Wilcoxon rank sum test with an absolute log2-fold change (FC) ≥ 0.5 and a p value < 0.05, including 193 upregulated genes in the PT group and 75 upregulated genes in the P group. C. GO enrichment analysis of DEGs in platelets. The color of the bubble indicates the Bonferroni-adjusted p value of the GO term, and the size of the bubble signifies the GeneRatio associated with a term. D. Volcano plot showing differentially expressed proteins (DEPs) between the PT and P groups. A total of 131 DEPs were identified using a fold change > 1.5 and a Q value < 0.05, including 79 upregulated proteins in the PT group and 52 upregulated proteins in the P group. E. KEGG enrichment analysis of DEPs. The color of the bubble indicates the -log10 (p value) of the KEGG terms, and the size of the bubble signifies the GeneRatio associated with a term.

Table S1. Clinical characteristics of 8 PNH patients

Clinical feature	PT1	PT2	P1	P2	P3	P4	P5	P6
Sex	Male	Female	Male	Female	Male	Female	Female	Male
Age(years)	36	54	36	20	46	28	31	51
RBC ($\times 10^{12}$/L)	2.58	1.9	1.97	2.28	2	2.17	2.49	2.82
WBC ($\times 10^{9}$/L)	3.49	3.72	3.45	5.47	2.89	7.52	2.54	6.06
HGB (g/L)	83	59	57	58	70	67	67	88
PLT($\times 10^{9}$/L)	34	70	89	78	139	219	209	157
Ret%	6.4	4.52	6.13	2.94	34.73	22.85	7.48	15.36
LDH (U/L)	778	678	982	291	4053	4410	1838	1664
TBIL (μmol/L)	18.9	19.8	20.1	8.2	35.8	82.1	34.5	49.2
IBIL (μmol/L)	12	11.4	14.3	6	25	74.1	26.3	33.3
PT (s)	13.9	14.5	10.1	12.4	10.7	10.6	12.8	11.3
PT-INR	1.27	1.32	0.92	1.13	0.98	0.97	1.17	1.03
APTT (s)	33.8	26.9	20.8	27.1	21.9	24.3	24	25.9
TT (s)	17.1	16.7	20.9	49.7	17.7	20.4	19.6	23.4
FIB (g/L)	4.44	5.52	2.43	0.6	3.83	2.57	4.46	2.01
D-Dimer (ng/ml)	3220	5217	633	108	2146	788	2231	945
$CD59^{-}$Erythrocyte (%)	8.46	12.15	32.13	7.75	97.58	42.56	86.49	48.87
$CD59^{-}$Granulocyte (%)	85.08	83.46	74.85	43.13	93.51	99.3	83.35	91.27
$CD14^{-}FLAER^{-}$ (%)	79.71	75	79.86	39.14	98.55	92.5	83.81	97.57
$CD24^{-}FLAER^{-}$ (%)	85.6	85.07	76.94	40.91	99.47	94.9	98.46	99.4

(RBC: red blood cell, WBC: white blood cell, HGB: hemoglobin, PLT: platelet, Ret: reticulocyte, LDH: lactic dehydrogenase; TBIL: total bilirubin; IBIL: indirect bilirubin; PT: Prothrombin Time, PT-INR: Prothrombin Time International Normalized Ratio, APTT: Activated Partial Thromboplastin Time, TT: Thrombin Time, FIB: Fibrinogen, FLAER: Fluorescently Labeled Aerolysin)

Table S2. Annotation information of mutant gene in PT group

Gene	PT1	PT2	Alt	Func	Exonic Func
ZNF471	0/1:0.54:31:37:68	0/1:0.54:41:49:90	G	exonic	nonsynonymous SNV
SLC35G4	0/1:0.17:79:16:95	0/1:0.25:46:15:61	A	exonic	nonsynonymous SNV
REG3A	0/1:0.12:60:8:68	0/1:0.16:80:15:95	T	exonic	nonsynonymous SNV
MUC17	0/1:0.12:838:117:955	0/1:0.12:715:101:816	A	exonic	nonsynonymous SNV
	0/1:0.12:837:117:954	0/1:0.13:698:101:799	A	exonic	nonsynonymous SNV
ASB1	0/1:0.47:63:57:120	0/1:0.41:95:65:160	T	exonic	nonsynonymous SNV
AHNAK2	0/1:0.09:675:68:743	0/1:0.08:622:57:679	A	exonic	nonsynonymous SNV
	0/1:0.08:669:62:731	0/1:0.08:618:53:671	C	exonic	nonsynonymous SNV
MAP3K2	0/1:0.50:43:43:86	0/1:0.41:44:31:75	C	exonic	nonsynonymous SNV
ANKRD36	0/1:0.15:63:11:74	0/1:0.13:71:11:82	G	exonic	nonsynonymous SNV
	0/1:0.17:91:18:109	0/1:0.15:82:15:97	-	exonic	frameshift deletion
KCNH6	0/1:0.51:163:172:335	0/1:0.48:142:132:274	A	exonic	nonsynonymous SNV
MUC19	0/1:0.10:71:8:79	0/1:0.10:85:9:94	A	exonic	nonsynonymous SNV
HCAR3	0/1:0.11:425:54:479	0/1:0.22:339:93:432	C	exonic	nonsynonymous SNV
MUC4	0/1:0.24:622:197:819	0/1:0.23:469:143:612	-	exonic	nonframeshift deletion
KRTAP10-12	0/1:0.16:174:33:207	0/1:0.19:170:40:210	C	exonic	nonsynonymous SNV
DNAH3	0/1:0.61:63:98:161	0/1:0.44:87:67:154	G	exonic	nonsynonymous SNV

Materials & Methods

1. Patients and clinical samples

Eight patients with classic PNH who were admitted to the Department of Hematology, Tianjin Medical University General Hospital, from September 2022 to July 2023, were enrolled in this study. There were 4 males and 4 females, and the median age was 36 years (range, 20–54 years). All patients were diagnosed in accordance with international PNH Study Group criteria. Two of these patients had acute thrombotic events, both of which were the first thrombotic events after the diagnosis of PNH, and the thrombotic site was the mesenteric vein. The clinical characteristics of the 8 PNH patients are provided in Table S1. Standard treatment involves glucocorticoids to control hemolysis, symptomatic blood transfusion, and anticoagulant therapy with low molecular weight heparin in thrombotic patients. None of these patients had received prophylactic antithrombotic therapy, complement inhibition therapy or hematopoietic stem cell transplantation. We detected CD59 and FLAER expression in peripheral blood to evaluate the proportion of PNH clones in patients. This study protocol was approved by the Ethics Committee of Tianjin Medical University General Hospital. All study subjects or family representatives were informed and signed an informed consent form.

2. Whole Exome Sequencing (WES)

Library preparation: 1 mL of EDTA anticoagulant peripheral blood from PNH patients were collected, and DNA was extracted using a DNA extraction kit (DP304; TIANGEN). Qubit 3.0 was used to accurately quantify the DNA concentration, and the DNA content in all samples was greater than 0.5 μg. The exome sequences were efficiently enriched from 0.4 μg genomic DNA using Agilent SureSelect Human All Exon V6 (Agilent USA, Catalog #: 5190-8864) according to the manufacturer's protocol. Qualified genomic DNA was randomly cleaved into 180–280bp by Covaris LE220R-plus (Covaris, USA). Then, DNA fragments were end repaired and phosphorylated, followed by A-tailing and ligation at the 3'ends with paired-end adapters. DNA fragments with ligated adapter molecules on both ends were selectively enriched in a PCR reaction. After PCR reaction, libraries hybridize with liquid phase with biotin labeled probe, then use magnetic beads with streptomycin to capture the exons of genes. Captured libraries were enriched in a PCR reaction to add index tags to prepare for sequencing. Products were purified using AMPure

XP system (Beckman Coulter, Beverly, USA), libraries were analyzed for size distribution by Agilent 2100/Agilent 5400 system (AATI) (Agilent, USA) and quantified by real-time PCR (Life Technologies, USA) (1.5 nM). The qualified libraries were pooled and sequenced on Illumina platforms, according to effective library concentration and the data amount required.

Bioinformatics Analysis： An average of about 10Gb raw data (fastq) as input was generated for each sample by Illumina sequencers. Firstly, the Paired-end reads was performed quality control by FASTQC version 0.11.5. Secondly, Burrows-Wheeler Aligner(BWA) version 0.7.15 was used to align sequencing reads to the reference genome GRCh38. SAM format files were generated by BWA. Thirdly, the SAM format files were further processed to BAM files using Samtools version 1.3.1 ,then removing duplicates by the picard function of GATK. After these processes, variant calling was performed by GATK version 4.2.6.1 and the VCF file was generated. Finally, we used an in-house software to annotate the variants from the VCF file and integrate information from multiple databases. The final variant is used for advanced downstream analysis.

3. Single-cell RNA sequencing (scRNA-seq)

Library preparation: 5ML of EDTA anticoagulant peripheral blood from PNH patients were collected to separate mononuclear cells with lymphocyte separation medium (Amersham Bioscience, UK). Considering the characteristics of PNH, in addition to mononuclear cells, we also retained granulocytes. Cells were washed with sterile phosphate-buffered saline, and the cell suspension concentration was adjusted to 300-600 cells /μL. Cell viability was analyzed with Countstar Rigel S2. Sequencing analysis was carried out after passing quality inspection. The cell suspension was loaded into Chromium microfluidic chips with 5’ v2 chemistry (P3, P4) or 3’ v3 chemistry (PT1, PT2) and barcoded with a 10× Chromium Controller (10X Genomics). RNA from the barcoded cells was subsequently reverse-transcribed and sequencing libraries constructed with reagents from a Chromium Single Cell 5’ v2 reagent kit (10X Genomics, CG000424 Rev C) or a Chromium Single Cell 3’ v3 reagent kit (10X Genomics, CG000315 Rev E) according to the manufacturer’s instructions. Sequencing was performed with Illumina (NovaSeq 6000) according to the manufacturer’s instructions (Illumina).

Bioinformatics Analysis：The Cell Ranger software pipeline (version 7.0.1 or 7.1.0) provided by 10xGenomics was used to demultiplex cellular barcodes, map reads to the genome and transcriptome using the STAR aligner, and down-sample reads as required to generate normalized aggregate data across samples, producing a matrix of gene counts versus cells. A total of 9,280 cells (6,134-13,100) were sequenced in each sample, with an average of 1,360 genes detected per cell. Then, we processed the unique molecular identifier (UMI) count matrix using the R package Seurat (version 4.3). Cells with more than 7,500 or less than 200 detected genes as well as those with a mitochondrial transcription ratio >5% were discarded. Cell doublets were identified using R packages “scDblFinder” and then discarded. ‘LogNormalize’ normalization methods was performed in Seurat on the filtered matrix to obtain the normalized count. Batch effects derived from technical and biological covariates were corrected using harmony version 1.0.3. Next, the graph-based clustering algorithm of Louvain after computing the nearest neighbor graph was used to partition the cells. The resulting clusters were visualized in a 2-D embedding produced by t-Distributed Stochastic Neighbor Embedding (t-SNE). Finally, the resulting coordinates of t-SNE and cluster tags for each cell were assigned to the expression matrix for downstream analysis. Clusters were identified using the community identification algorithm as implemented in the Seurat "FindClusters" algorithm, and annotated based on marker genes. Considering that some PNH patients received red blood cell transfusions before sampling, red blood cells were removed after cell annotation was completed, 35,949 cells were included in subsequent analysis. The cells differentially expressed genes (DEGs) in the two groups was analyzed by “FindMarkers”, and the parameter of cells minimum proportion was 25%. Genes with an expression |log2FoldChange|>0.5 and p_val<0.05 were considered differentially expressed. The cell types between conditions were ranked using Augur tool with default parameters. R packages “clusterProfiler” was used to perform biological function enrichment analysis.

4. Isobaric Tags for Relative and Absolute Quantitation (iTRAQ) -based proteomics

iTRAQ-LC-MS/MS: Plasma was isolated from 5ML EDTA anticoagulant peripheral blood of PNH patients. Proteominer kit was used to remove the high abundance and collect the protein solution. The proteins were reduced and alkylated using 10 mM DTT and 55 mM iodoacetamide,

and Bradford method was used to quantify the protein concentration. Subsequently, proteins were trypsinised, desalted, and freeze dried. After the peptide was labeled with iTRAQ labeling reagent, 20μg mixed samples were separated on a Gemini C18 column (Phenomenex, Torrance, CA, USA) by Shimadzu LC-20AD liquid chromatography system(Shimadzu, Kyoto, Japan). The elution peak was monitored at 214nm wavelength and one component was collected every 3.15min. 20 components were obtained by combining with the chromatographic elution peak diagram, and then frozen drained. The peptide samples were dissolved with mobile phase A(2% ACN, 0.1% FA), centrifuged at 20000 g for 10min, and the supernatant was extracted and separated by Thermo UltiMate 3000 UHPLC (Thermo Fisher Scientific). Finally, the separated peptides were ionized by nanoESI source and detected using a Q Exactive HF-X Hybrid Quadrupole-Orbitrap mass spectrometer (Thermo Fisher Scientific) operated in data dependent acquisition (DDA) mode. The above iTRAQ experimental procedures were completed by BGI Genomics (Shenzhen, China).

Bioinformatics Analysis: The raw MS/MS data were converted into MGF format by Proteome Discoverer (Thermo Fisher Scientific), and aligned with the NCBI database using Mascot v2.3.02 identification software. A total of 3717 peptides and 761 proteins were identified after QC and filtering (1% FDR). IQuant software (BGI Genomics) was used to quantify proteins and screen for significantly differentially expressed proteins (DEPs) between the two groups (fold change> 1.5 and Q value < 0.05). Subsequently, DEPs were analysed using Gene Ontology (GO) aggregation analysis, Kyoto Encyclopedia of Genes and Genomes (KEGG) pathway analysis, and functional annotation.
